# Swedish doctors choice of medical speciality and associations with cultural capital and perceived status: a cross-sectional study

**DOI:** 10.1186/s12909-019-1691-y

**Published:** 2019-07-04

**Authors:** Caroline Olsson, Hans Järnbert-Pettersson, Sari Ponzer, Marie Dahlin, Tomas Bexelius

**Affiliations:** 1 Department of Clinical Science and Education, Karolinska Institutet, Forskningscentrum, Sjukhusbacken 10, Södersjukhuset, SE-118 46 Stockholm, Sweden; 2 Department of Clinical Science and Education, Södersjukhuset, Karolinska Institutet, Stockholm, Sweden; 3 Centre for Psychiatry Research, Department of Clinical Neuroscience, Karolinska Institutet & Stockholm Health Care Services, Stockholm County Council, S:t Görans Hospital, Vårdvägen 1, SE-112 81 Stockholm, Sweden; 4 Department of Women’s and Children’s Health, Childhood Cancer Research Unit and Department of Medical Epidemiology and Biostatistics, Karolinska Institutet, Stockholm, Sweden

**Keywords:** Specialty choice, Cultural capital, Prestige, Status, Medical field

## Abstract

**Background:**

Many western countries have problems recruiting and retaining medical specialists. In Sweden there is a lack of primary care doctors and psychiatrists. Despite much research on the topic the shortage remains. We therefore set out to analyse choice of medical speciality using Bourdieu’s theoretical concepts; cultural capital, social background and perceived status.

**Methods:**

A cross-sectional questionnaire-based study of 399 alumni from the Medical School at Karolinska Institutet, Stockholm was performed. The response rate was 72% (*n* = 286); 262 of the respondents were in training to become specialists. Specialties were categorized as primary care, psychiatry, internal medicine, and surgical and hospital service specialties. To study the associations between medical specialties and cultural capital, we used multinomial regression analyses. Variables that showed a significant association with medical specialties were included in an adjusted multivariable model. These results were presented as odds ratios: the odds that a particular speciality is chosen in comparison to a choice of surgery as a speciality, based on perceptions of *high status*.

**Results:**

The results were analysed using Bourdieu’s theoretical concepts of cultural capital, in the form of educational capital and social prestige. We found distinctive differences in perceived status for the examined speciality groups, ranging from 70% high status for surgery down to 6% high status for geriatrics and primary care. Perceived status was also associated with respondents’ own speciality choice, presented as an odds ratio. Our data did not show any associations between speciality choice and educational capital. We also included sociodemographic data.

**Conclusion:**

The field of medicine is according to Bourdieu an arena for power struggles. Knowledge of the distinctive differences in perceived status between medical specialties can be an asset particularly in relation to recruitment and retainment of specialist doctors. Our results could be used to identify specialities where perceptions of low status may be contributing to a shortage of specialists.

## Background

There is a need for a greater understanding of factors such as social background and educational capital which underlie doctors’ choice of speciality. Previous findings show associations between speciality choice and work related factors such as workload, type of patient relations and respondents interest in specific diseases [1]. In a previous article we examined the association between doctors’ speciality choice and personality traits, showing that surgeons had a higher degree of conscientiousness than other speciality groups [2]. As specialist shortages only occur within certain specialities – namely geriatrics and primary care - it is important to know why [3]. In this paper we have therefore chosen to focus on other factors that can be related to choice of speciality and by doing so we wish to contribute to a greater understanding of how to solve the problem of shortages.

Bourdieu’s theoretical framework is useful in better understanding the underlying characteristics that influence the individual in her or his choice of speciality. It challenges both the idea of choice as something entirely rational and the idea that individuals are completely trapped within social structures [4]. In this study we investigate doctors’ speciality choice by applying Bourdieu’s concepts of medical field, cultural and symbolic capital, in the forms of inherited and acquired educational capital, and perceived prestige of different medical specialties [5].

Bourdieu’s concept of educational capital has been used to a large extent in educational research, mainly focusing on children in younger years and on access to higher education and admission to medical school [6–8]. It has also to a certain extent been used to investigate achievement of students within the higher education system [9] and how students from socio-disadvantaged backgrounds have adapted to medical school and its culture [6, 10]. Brosnan et al. showed that Bourdieu’s concepts were useful in describing power relations within the medical field by analysing differences in capital for medical schools in the U.K. [6]. Cleland et al. established that cultures in medical school impact on speciality preferences [11]. There are previous studies showing that perceived status or prestige is associated with speciality choice [12–14]. Prestige and status are essential in Bourdieu’s theoretical framework. It is by struggling for assets and prestige within a field that power is distributed. Investigating how doctors rank different specialties according to perceptions of status contributes to an understanding of how the medical field is constructed [4, 15].

### Theoretical framework

This article draws on the theoretical framework of the French anthropologist and sociologist Pierre Bourdieu whose concepts have been developed and used in educational research [8, 16, 17]. One of the benefits in using Bourdieu’s concepts when analysing doctors’ career choices is that it makes it possible to combine a structural analysis with an individual perspective. According to Bourdieu the social world is ordered by structures and individuals are born into social positions, however they are also agents with possibilities to move up and down in the social hierarchy [18].

One of the more important concepts in Bourdieu’s educational sociology is ‘field’, and it has been argued that the medical world could be defined as a field, the field of medicine. A field can be described as the context in which agents act and invest to pursue their medical career in competition with others [19–21]. Within the field of medicine, other doctors and their positions are more important to you in your professional life than any other profession or social position. The agents within a field fight over assets and positions using different forms of capital [19].

Bourdieu used three main forms of capital when analysing the social order of a field; economic capital, social capital (e.g. networks, groups) and cultural capital. These forms of capital can be transformed into symbolic capital. Symbolic capital is defined within a specific field, is constituted of what is recognized as important in that field, and is therefore indicative of prestige or high status [14, 20]. Symbolic capital is useful for analysing specific values of prestige and status within medicine [4, 22, 23].

Educational capital is a form of cultural capital and can be divided into two subgroups – inherited and acquired educational capital. The former refers to inherited assets for instance being born into a well-educated family. The latter refers to assets acquired through personal investment in the educational system. Grades, courses and exams are examples of acquired educational capital [24, 25]. Inherited and acquired educational capital are related, for instance, someone born into a family with high cultural capital is more likely to develop skills and knowledge that are useful in school and consequently – in most cases – receive higher grades etc. [26]. Access to medical school requires a high level of acquired educational capital. In Sweden there are two ways of qualifying for admission to medical school, either through the upper secondary school programme or through the National Admission Test (SweSat). As elsewhere, admission requires high grades or scores. Previous research shows that access to medical education is associated with social background and educational capital [6] .

The aim of this study was threefold. Firstly, to investigate associations between medical doctors’ educational capital and their choice of speciality. Secondly to investigate doctors’ perceptions of status regarding specialties. Thirdly, to analyse these associations by applying some of the theoretical concepts developed by Pierre Bourdieu.

## Methods

### Study design and setting

We performed a cross-sectional study based on a postal questionnaire sent to former medical students at Karolinska Institutet, Stockholm. The cohort had participated in previous research studies while still in medical school. The original data collection occurred in three phases during the years 2001–2002, 2004–2005 and 2006. Four hundred twenty-six respondents who had participated in at least one of the previous surveys were eligible for inclusion in the present study. Out of these, 27 were excluded owing to non-traceability (*n* = 10), not working as a doctor (n = 1) or being registered as living abroad (*n* = 16). Hence, 399 (94%) respondents were traceable and contacted. Age, gender and information regarding qualification for admission to medical school was based on the original data collection [27, 28]. The paper questionnaire was posted to 399 medical school alumni during the spring of 2013. Non-respondents were sent a reminder during late spring, 2013, with a new paper questionnaire enclosed. Two hundred eighty-nine were completed by the respondents, resulting in a response rate of 72%. All in all, 21 doctors (7%) reported that they had not yet started their specialist training and, for 6 individuals, data on speciality choice was missing, leaving 262 participants (90.6% of the respondents) for analyses regarding speciality choice. The questionnaire included questions regarding speciality choice, current working conditions, previous education, personality and mental health.

### Medical specialties

Our outcome was defined as ‘type of medical speciality’. We categorized the specialties into the following five groups based on The National Board of Health and Welfare’s main categories: primary care, internal medicine specialties (including paediatrics, geriatrics, cardiology, neurology, gastroenterology, dermatology, infectious diseases, oncology, haematology, rheumatology and allergology), surgical specialties (including thoracic surgery, trauma surgery, plastic surgery, neurosurgery, otorhinolaryngology, ophthalmology and urology, as well as anaesthesiology, emergency medicine, orthopaedics, obstetrics and gynaecology), psychiatry (including child psychiatry) and hospital service specialties (including radiology, clinical pathology, clinical genetics, clinical chemistry, forensic medicine and occupational and environmental medicine) [2].

### Sociodemographic variables

Exposure variables were different aspects of social background and symbolic capital. Social background refers to parental occupation and was based on the parent with the highest ranking profession. The coding was done in accordance with the Swedish national administrative agency for statistics (Statistics Sweden) and consisted of the following categories; working class, lower to middle class and upper middle class. Age was dichotomized, to more than or less than 37 years of age, to achieve two balanced categories (median value 37 and mean value 38 years). Foreign background was defined as if the respondent himself/herself or both parents were born outside of Sweden, and divided into two categories – Swedish and foreign.

### Symbolic capital in form of inherited and acquired educational capital

With Bourdieu’s theoretical concepts in mind we created questions that would generate a picture of the importance of symbolic capital and measure to what extent doctors with different specialties had gathered such capital. To measure *inherited educational capital* the following two variables were used: Parents’ highest level of education (based on the parent with the highest educational level and coded in accordance with Swedish Higher Education Authority and Swedish Council for Higher Education) [29] and a yes/no question to find out if the respondents had at least one parent that was a medical doctor. To measure the respondents *acquired educational capital*, we used several variables: Type of school (public or private, where private indicates higher educational capital), Upper secondary programme (natural science, social science, technical science/other, where natural science indicate the highest educational capital) [30], Grades (In Sweden there have been several changes in the grading system and in the research population there are three different upper secondary grade scales represented). The different grading systems were harmonised through recalculation based on standards from Swedish Higher Education Authority and Swedish Council for Higher Education [31] and the used scale ranges from 0.0 to 20.0. In accordance with this harmonisation and previous research we coded two groups to distinguish those who had the highest grades, since high grades are an important asset in educational capital, from the rest with 20.00–19.00 as one category and 18.99 and lower as the other category [32]. In a similar way SweSat results were coded into two categories to distinguish those who had the highest results from the rest. The national scale ranges from 0.0–2.0 and we divided them into two categories (2.0–1.8 and 1.7–0.1). Previous higher education was coded into three categories: none, up to 3 years, more than 3 years. A question regarding previous research education was dichotomised into yes or no answers.

### Symbolic capital in form of perceived status

To measure symbolic capital in the form of *prestige* and *status* we used a Likert-scale type question ranging from very high [1] to very low [6] perceived status of different medical specialties. Since we were interested in measuring the influence of perceived high status we coded the two highest scores in the Likert scale [1, 2] as high status and the rest [3–6] as low status.

### Statistics

Statistical Package for Social Sciences (SPSS v.22.0) was used for statistical analyses. Descriptive data was stratified into five speciality groups: primary care, internal medicine specialties, surgical specialties, psychiatry and hospital service specialties. For comparisons of the proportional size of the groups and other background variables, we used Fischer’s Exact test (Monte Carlo), with a significance level of *p* < 0.05. To study the associations between medical specialties (outcome variable, categorized in five groups) and different exposure variables of symbolic capital, we used multinomial regression analyses. First we performed a univariable analysis to study the association for each of the exposure variables and medical speciality one at a time. Secondly, variables that showed a significant association with medical specialties (e.g. perceived high status) were included in an adjusted multivariable model, in order to analyse if there were any associations between perceived *high status* and respondent’s chosen speciality. These results are presented as odds ratios (ORs) with 95% confidence intervals (CIs) as an estimate of the odds that a certain specialty is chosen compared to surgery with regard to perceived high status. In addition, we decided a priori to include personality traits, age and gender in the adjusted model. A *p*-value of < 0.05 was considered significant.

### Ethics committee approval

The research was performed in accordance with the Helsinki Declaration. The questionnaire was anonymous and voluntary. Informed consent was obtained from all participants. The Regional Ethical Review Board in Stockholm approved the study.

## Results

### Sociodemographic data

37% (*n* = 97) of the respondents were men, see Table 1. The highest proportion of men was in the hospital service group, 47% (n = 9), followed by surgeons, 44% (*n* = 37) (*p* = 0.18). The lowest proportion of men was in the psychiatry group with 19% (*n* = 4) men. Only 10 (4%) of the respondents came from a working class background, 71 (28%) came from lower to middle class background and 171 (68%) from an upper middle class background (*p* = 0.73). Completion of specialist training was the only basic characteristic that differed significantly by speciality group (*p* = 0.04): More surgeons had completed their training compared to doctors in other specialties (32% compared with the average of 22%) at the time of data collection (Table 1).

**Table 1:** Basic characteristics of the participants, by chosen speciality

	Total		Surgical		Primary care		Internal medicine		Psychiatry		Hospital service		
	n	%	n	%	n	%	n	%	n	%	n	%	*p*-value
Gender													
Female	165	63	47	56	43	64	48	68	17	81	10	53	0.181
Male	97	37	37	44	24	36	23	32	4	19	9	47	
Age													
Up to 37	144	55	51	61	33	49	38	53	9	43	13	68	0.326
From 38	118	45	33	39	34	51	33	46	12	57	6	32	
Social background													
Working class	10	4	4	5	2	3	2	3	1	5	1	5	0.733
Lower to middle class	71	28	22	27	15	23	25	37	5	24	4	21	
Upper middle class	171	68	55	68	47	73	40	60	15	71	14	74	
Ethnic background													
Swedish	228	88	74	89	61	92	58	83	18	86	17	94	0.450
Foreign	30	12	9	11	5	8	12	17	3	14	1	6	
Marital status													
Married/Registered partner	142	57	42	52	44	68	37	57	12	57	7	37	0.296
Partner/Not married	88	35	32	39	18	28	23	35	6	29	9	47	
Single	21	8	7	9	3	5	5	8	3	14	3	16	
Status of residency training													
Ongoing	202	78	55	68	52	78	58	82	19	90	18	95	0.039*
Completed	57	22	26	32	15	22	13	18	2	9	1	5	

Values with * denote statistically significant differences, *P* < 0.05, using Fischer’s exact test for comparisons between groups

### Symbolic capital in the form of inherited and acquired educational capital

The numbers and percentages of the different variables to measure educational capital, stratified by speciality type, are shown in Table 2. We found no significant associations between doctors’ speciality choice and inherited or acquired educational capital (therefore no regression analysis is presented for educational capital).

**Table 2:** Distribution of educational capital within different speciality groups

	Total		Surgical		Primary care		Internal medicine		Psychiatry		Hospital service		
	n	%	n	%	n	%	n	%	n	%	n	%	*p*-value
Parents’ highest education													
Secondary school	46	18	13	16	11	17	14	20	5	24	3	17	0.878
University	170	66	57	69	47	71	43	61	12	57	11	61	
Doctoral studies	41	16	12	15	8	12	13	19	4	19	4	22	
Physician parent													
Yes	165	76	56	77	44	80	44	77	12	67	9	69	0.751
No	51	24	17	23	11	20	13	23	6	33	4	31	
Type of school													
Public school	231	88	74	88	63	94	61	86	17	81	16	84	0.334
Private school/other	31	12	10	12	4	6	10	14	4	19	3	16	
Upper secondary programme													
Natural science	171	66	54	64	45	68	49	70	10	48	13	72	0.175
Social science	49	19	16	19	11	17	10	14	10	48	2	11	
Technical/other	39	15	14	17	10	15	11	16	1	5	3	17	
Grades													
20.0–19.0	106	42	38	46	25	40	29	41	8	40	6	35	0.901
18.99–0.0	146	58	44	54	38	60	41	59	12	60	11	65	
SweSat results													
2.0–1.8	151	66	48	70	39	63	41	64	15	83	8	47	0.216
1.7–1.0	79	34	21	30	23	37	23	36	3	17	9	53	
Previous higher education													
None	103	39	31	37	28	42	27	39	5	24	12	63	0.075
Up to 3 years	110	42	40	48	23	34	30	43	14	67	3	16	
3 years or more	48	18	13	15	16	24	13	19	2	9	4	21	
Research education													
Yes	35	13	74	88	62	92	57	80	19	90	15	79	0.199
No	227	87	10	12	5	7	14	20	2	9	4	21	
Total	262	100	84	100	67	100	71	100	21	100	19	100	

Monte Carlo and Fischer’s exact test were used for comparisons between groups

In total, 41 (16%) of the doctors had at least one parent with a PhD; of the Primary care doctors, 8 (12%) reported having a parent with a PhD. The corresponding proportion among hospital service specialists was 22% [4], but the difference was not statistically significant (*p* = 0.88). Twenty four per cent of the respondents (*n* = 51) had at least one parent that was a medical doctor. The most common upper secondary school programme amongst all doctors was the natural science programme, ranging from 65 to 70%, except for psychiatrists where only 48% (*n* = 10) had completed the natural science programme (*p* = 0.17). Surgeons had the highest proportion in the “high grades” group with 46% (*n* = 38) compared to the average that had 42% (*n* = 106) and hospital service group that had the fewest high grades with 35% (*n* = 6) (*p* = 0.90). Results within the highest range at the SweSat had been achieved by 151 (66%). The psychiatrists had the highest proportion of high SweSat results with 15 (83%); the lowest proportion 8 (47%) was found for the hospital service group (*p* = 0.21) (Table 2).

### Symbolic capital in form of perceived status

There were distinctive differences in status as perceived by the respondents, ranging from 69% of respondents attributing high status to surgery down to 6% attributing high status to laboratory specialties and to geriatrics, regardless of the respondent’s own speciality. Results of perceived status of different specialties are shown in Table 3. Surgery scored the highest result, where 186 (69%) of all doctors ranked surgery as *high status* (*p* = 0.00), followed by neuro specialities, ranked by 137 (51%) as *high status* (p = 0.00). In total, the lowest scores for perceived *high status* were given to the following specialities: laboratory, 16 (6%) (*p* = 0.20), geriatrics, 17 (6%) (*p* = 0.23) and psychiatry, 18 (7%) (*p* = 0.27). Among the surgeons 66 (83.5%) ranked surgery as having *high status*. Only 4 (5%) of the surgeons ranked primary care as having *high status*. In contrast, among the primary care speciality, 16 (25%) ranked primary care as having *high status* (Table 3).

**Table 3:** Distribution of high and low perceived status of different specialities, by chosen speciality

Type of chosen speciality													
	Total		Surgical		Primary care		Internal medicine		Psychiatry		Hospital service		
Perceived status for:	n	%	n	%	n	%	n	%	n	%	n	%	*p*-value
Primary Care													
High	41	15	4	5	16	25	7	10	8	42	3	17	0.000*
Low	229	85	74	95	49	75	60	90	11	58	15	83	
Surgery													
High	186	69	66	83.5	43	67	40	60	8	42	12	67	0.001*
Low	84	31	13	16.5	21	33	27	40	11	58	6	33	
Geriatrics													
High	17	6	2	2.5	7	11	4	6	2	10	1	6	0.226
Low	253	94	77	97.5	57	89	63	94	17	89	17	94	
Internal medicine													
High	127	47	23	29	42	66	29	43	11	58	7	39	0.000*
Low	143	53	56	71	22	34	38	57	8	42	11	61	
Psychiatry													
High	18	7	3	4	5	8	4	6	3	16	2	11	0.273
Low	252	93	76	96	59	92	63	94	16	84	16	89	
Laboratory													
High	16	6	3	4	7	11	2	3	2	11	1	6	0.197
Low	247	94	73	96	55	89	65	97	16	89	17	94	
Imaging/radiology													
High	47	17	17	22	12	19	8	12	3	16	4	22	0.575
Low	222	82	61	78	52	81	59	88	16	84	14	78	
Neuro-specialities													
High	137	51	25	33	44	69	30	45	11	58	10	56	0.001*
Low	130	49	51	67	20	31	37	55	8	42	8	44	

Values with * denote statistical significant result, *P* < 0.05, using Fischers exact test to compare between groups

Table 4 shows the results of how perceived *high status* of each speciality associates with respondent’s own speciality choice over the choice of surgery as reference, e.g. if a respondent perceived primary care as having *high status;* odds were 6.04 times higher that he or she had chosen primary care instead of surgery (crude analysis). After adjustment for personality traits, age and gender the odds were of the same order, 7.46 times higher.

**Table 4:** Associations between perceived high status for eight different specialities and respondent’s own speciality choice

Respondent’s own choice of specialty						
High Status for:	Surgery OR (95% CI)		Primary care OR (95% CI)	Internal medicine OR (95% CI)	Psychiatry OR (95% CI)	Hospital-based service OR (95% CI)
Primary care	Crude	Ref.	6.04 (1.91–19.15)	2.16 (0.60–7.72)	13.45 (3.46–52.27)	3.70 (0.75–18.26)
	Adjusted	Ref.	7.46 (2.21–25.24)	2.39 (0.63–8.99)	18.21 (3.82–86.74)	5.40 (1.02–28.60)
Surgery	Crude	Ref.	0.40 (0.18–0.89)	0.29 (0.13–0.63)	0.14 (0.05–0.42)	0.39 (0.12–1.24)
	Adjusted	Ref.	0.35 (0.15–0.84)	0.22 (0.09–0.52)	0.07 (0.02–0.29)	0.47 (0.13–1.80)
Geriatrics	Crude	Ref.	4.73 (0.95–23.61)	2.44 (0.43–13.78)	4.53 (0.59–34.46)	2.26 (0.19–26.43)
	Adjusted	Ref.	5.28 (0.95–29.33)	2.16 (0.35–13.20)	4.92 (0.51–47.76)	4.00 (0.30–53.81)
Internal medicine	Crude	Ref.	4.65 (2.29–9.44)	1.86 (0.94–3.69)	3.35 (1.19–9.40)	1.55 (0.53–4.49)
	Adjusted	Ref.	4.52 (2.13–9.60)	1.59 (0.76–3.32)	2.17 (0.66–7.13)	2.10 (0.65–6.83)
Psychiatry	Crude	Ref.	2.15 (0.49–9.35)	1.61 (0.35–7.46)	4.75 (0.88–25.71)	3.17 (0.49–20.52)
	Adjusted	Ref.	2.19 (0.46–10.40)	1.47 (0.30–7.30)	5.21 (0.78–34.83)	4.42 (0.61–32.15)
Laboratory	Crude	Ref.	3.10 (0.77–12.52)	0.75 (0.12–4.62)	3.04 (0.47–19.72)	1.43 (0.14–14.62)
	Adjusted	Ref.	3.78 (0.85–16.89)	0.83 (0.12–5.44)	3.67 (0.47–28.60)	2.28 (0.20–25.57)
Imaging/radiology	Crude	Ref.	0.83 (0.36–1.89)	0.49 (0.19–1.21)	0.67 (0.17–2.58)	1.02 (0.30–3.52)
	Adjusted	Ref.	0.85 (0.35–2.03)	0.48 (0.19–1.25)	0.81 (0.19–3.47)	1.31 (0.35–4.89)
Neuro specialities	Crude	Ref.	4.49 (2.20–9.16)	1.65 (0.84–3.26)	2.80 (1.00–7.85)	2.55 (0.90–7.25)
	Adjusted	Ref.	5.14 (2.35–11.27)	1.52 (0.73–3.18)	1.80 (0.54–6.06)	4.20 (1.25–14.10)

The crude analyses are non-adjusted results between each of the ranked specialities and respondent’s own speciality compared with surgery as reference. Significant results; Primary care, Surgery, Internal medicine, Neuro specialities. Not significant: Geriatrics, Psychiatry, Laboratory, Imaging/radiology

Adjusted for personality traits, age and gender

Significant differences between respondent’s own speciality choice compared with surgery among the following outcomes: Primary care, Surgery, Internal medicine, Imaging/radiology, Neuro specialities. Not significant: Geriatrics, Psychiatry, Laboratory

## Discussion

There were no statistically significant differences in social background or the amount of educational capital between different medical specialists. There were, however, distinctive differences in perceived status for the different specialities and we can conclude that doctors perceived their own specialities as disproportionally high in status.

We know from earlier research that admission to higher education and to medical school is associated with social background and educational capital. We wished to investigate if differences in social background and educational capital were associated with choice of medical speciality, something that has not been previously investigated to any great extent. However, we found no significant associations between specialty choice, social background and educational capital. We assume that one important reason for this is that students entering medical school are a highly selective group. Social class and educational capital are high in all the specialties in our empirical data.

We also wanted to examine if doctors viewed specialties differently in terms of perceived status and to analyse associations with their own speciality of choice. For the present study we operationalized Bourdieu’s complex concepts, by “translating” them into questions or statements that were suitable for a questionnaire. This is complex and there are no examples in the literature. During the process of creating the questionnaire we had elaborate discussions about the ideal design of questions. We found distinctive differences between speciality choice and perceived status. Overall surgery scored *high status* from two thirds of the study population compared to geriatric, psychiatry and laboratory specialties that all scored around 6% *high status*. Surgeons were also the group that gave more diverse scores regarding status when ranking their own and other specialties, ranking *surgery* highest compared to all of the rankings with 83.5% *high status*. On the other hand, only 2.5% of the surgeons gave *high status* to geriatrics and around 6% to psychiatry and laboratory specialties.

The respondents were, at the time of data collection, already working as medical doctors, most of them doing their specialist training. There is a risk in using a retrospective perspective. Respondents who have already made their choice and entered the culture of their speciality might give answers coloured by that experience. At the same time, using respondents who have made their choice is also a strength of this study. We know from previous research that medical students who have been asked about their future choices tend to change their minds, sometimes more than once, before making their actual choices [33]. The best way to examine choice of speciality might be to follow a cohort from when they are students to fully qualified specialists.

We conducted a cross-sectional study with a response rate of over 70%. One of the limitations in using a cross-sectional approach has to do with causality. We can establish associations between the respondents’ own speciality and perceived status; however we cannot rule out that once you belong to a certain speciality group, you become influenced by your perception about that specialty (or other specialties). Using a quantitative method has both advantages and limitations. On the one hand, it provides a chance to establish associations. On the other hand, examining choice is complex not easily captured adequately in a questionnaire. We are well aware of this limitation and in the future we will continue to examine specialist choice as a phenomenon using qualitative methods.

Another limitation is the risk of type two errors since the numbers in some cells are quite small. We present all our analyses for maximal transparency.

In a previous article we studied speciality choices and personality traits and we found significant associations, where Agreeableness was lower for surgeons than for doctors in internal medicine, hospital service and primary care. Conscientiousness on the other hand was higher for surgeons than for psychiatrists and hospital service physicians [2]. For this reason we also adjusted our status model for personality traits, gender and age. We found significant associations between doctors’ choice of speciality and perceived high status even after adjusting for those factors.

We have argued that the medical field is one where power relations exist and where doctors use their capital to gain a position within that field. We did not operationalise Bourdieu’s concept *habitus* directly in our investigation but it is central in understanding the power relations within a field. Habitus can be defined as systems of dispositions that enable individuals to act, think and navigate in the social world [20] or as Dhen and Eika put it “[Habitus is the] embodied mental structures directing our actions, practices and meaning” [4]. Bourdieu argues that fields, like the medical field, and individuals’ habitus are under constant change. We are born into social structures that shape habitus but the educational system and other experiences in life will develop your habitus [7]. We found that speciality choice is associated with perceived status and for Bourdieu habitus is shaped in relation to status and prestige. A better understanding of the power relations in the medical field creates an opportunity for medical schools and employers to work with the image of different specialities. Why is surgery considered to have high status whereas other specialties are ranked much lower? A better understanding of doctors’ speciality choice in relation to perceived status may provide ways to enable “promotion” of those specialties that face a shortage of professionals, by trying to change the image of low status specialities.

There is not much published research linking doctors’ speciality choice to Bourdieu’s theoretical framework [4, 6, 15]. This explorative study of doctors’ speciality choice using Bourdieuan concepts and perspectives should be seen as one contribution in need of complementary studies. In further studies of the medical field and its relation to doctors’ speciality choice, research methods with a qualitative approach should be applied, to get a greater understanding of how medical doctors’ habitus is developed, from childhood to being a medical specialist. It is a complex process and should involve questions about the respondents’ childhood, role models, school experiences and so forth. In other words, what is needed is a method that allows in-depth analyses about the mechanisms that make people invest in educational capital to the extent of becoming a medical specialist and what makes them prepared to compete over assets within the medical field.

## Conclusions

We found no significant associations between medical doctors’ speciality and inherited and acquired educational capital. Doctors, regardless of which speciality they have, possess high educational capital. We assume that one important reason for this is that they are already highly selected when they enter medical school. There are, however, differences in mean values for grades, result on the national admission test and for type of upper secondary programme. Our main findings were that there were distinctive differences in perceived status of different specialities, ranging from 69% high status for surgery and down to 6% high status for laboratory specialties and for geriatrics. In Bourdieu’s world prestige and status are essential to understanding the power relations in a field, like the medical field. Speciality choice and its relation to perceived status is therefore one important factor when analysing doctors’ positions and investments within the medical field.

## Data Availability

The dataset supporting the findings and the conclusions of this article are available.

## Abbreviations

SweSat: Swedish national admission test for higher education
